# Artificial Intelligence through Wireless Sensors Applied in Restorative Dentistry: A Systematic Review

**DOI:** 10.3390/dj12050120

**Published:** 2024-04-24

**Authors:** Carlos M. Ardila, Annie Marcela Vivares-Builes

**Affiliations:** 1 Basic Studies Department, School of Dentistry, Universidad de Antioquia UdeA, Medellín 050010, Colombia; 2 School of Dentistry, Institución Universitaria Visión de Las Américas, Medellín 050031, Colombia; anny.vivares@uam.edu.co

**Keywords:** wireless technology, artificial intelligence, dental technology, dental care

## Abstract

The integration of wireless sensors with artificial intelligence could represent a transformative approach in restorative dentistry, offering a sophisticated means to enhance diagnostic precision, treatment planning, and patient outcomes. This systematic review was conducted to pinpoint and assess the efficacy of wireless sensors in restorative dentistry. The search methodology followed the guidelines outlined by PRISMA and involved the utilization of prominent scientific databases. Following the final phase of evaluating eligibility, the systematic review included six papers. Five experiments were conducted in vitro, while one was a randomized clinical trial. The investigations focused on wireless sensors for cavity diagnosis, toothbrush forces, facial mask applications, and physiological parameter detection from dental implants. All wireless sensors demonstrated efficacy in achieving the objectives established by each study and showed the validity, accuracy, and reproducibility of this device. The investigations examined in this systematic review illustrate the potential of wireless sensors in restorative dentistry, especially in the areas of caries detection, dental implant systems, face masks, and power brushes. These technologies hold promise for enhancing patient outcomes and alleviating the workload of dental practitioners.

## 1. Introduction

Restorative dentistry is commonly perceived as the primary focus of dental practice, often associated with operative interventions to repair or replace teeth. However, it is a broader field that involves diagnosing and managing diseases affecting teeth and their supporting structures, followed by the restoration and rehabilitation of the dentition. This approach aims to address both functional and aesthetic needs. The necessity for restorative dentistry often arises from tooth damage due to various factors, such as caries, non-carious tooth surface loss, trauma, or developmental defects. This damage can occur at any stage of life, and the restorative strategies employed will be tailored to the individual’s specific circumstances [1].

Dental decay, gum diseases, injuries, and genetic disorders, coupled with aging, are significant contributors to partial tooth damage or complete tooth loss. This underscores the need for developing approaches aimed at managing these conditions and restoring lost tissues, which should not overlook the potential and significance of traditional techniques in restorative dentistry. These strategies encompass a diverse array of clinical procedures, leveraging both advanced technologies and high-performance biomaterials, along with time-tested traditional techniques, that closely resemble the lost natural tissues. These innovations, including traditional methods alongside technological advancements, spanning from digitalization to nanotechnology, have substantially enhanced the efficacy of dental care. They render restorative treatments more efficient, pain-free, less time-intensive, and more comfortable for patients compared to conventional methods [2].

Artificial intelligence involves the emulation of human intelligence in machinery, enabling them to think and learn similarly to humans. This incorporates a range of technologies, such as “machine learning, natural language processing, computer vision, and robotics”. Artificial intelligence can explore vast datasets, identify patterns, make decisions, and execute tasks that traditionally rely on human intelligence [3,4].

Wireless sensors, when integrated into the framework of artificial intelligence, serve as data collection tools that provide real-time information about a specific environment or system. These sensors can be strategically placed in various locations to monitor specific parameters, such as temperature, humidity, pressure, or motion. The data collected by these sensors are then transmitted wirelessly to an artificial intelligence system, where they are analyzed and processed using algorithms to extract meaningful insights or make informed decisions [5,6].

The integration of wireless sensors with artificial intelligence could represent a transformative approach in restorative dentistry, offering a sophisticated means to enhance diagnostic precision, treatment planning, and patient outcomes [7,8,9,10,11,12]. Wireless sensors, through their ability to continuously monitor various oral parameters, provide a wealth of real-time data that can be harnessed to inform clinical decision-making. Artificial intelligence algorithms, on the other hand, possess the capacity to evaluate massive quantities of complex information with remarkable speed and precision, thereby unlocking valuable insights that may not be readily apparent through conventional methods [7,9,11].

For instance, artificial intelligence algorithms can process the data from wireless sensors to identify early signs of dental caries [8,9,12], allowing for prompt intervention and preventive measures. Moreover, artificial intelligence can assist in treatment planning by analyzing patient-specific data to recommend the most suitable restorative materials, techniques, and procedures [7,8,9,10,11,12]. Additionally, artificial-intelligence-driven analytics can help predict the long-term success of restorative treatments [8,9,12], enabling dentists to tailor their approach based on individual patient needs and characteristics. In the field of restorative dentistry, various applications of artificial intelligence have been evaluated [13,14], apart from the application of wireless sensors. To fully harness the potential of wireless sensor methodologies in restorative dentistry, a comprehensive description of the functionality and constraints of wireless sensors is essential. Therefore, this systematic review was conducted to identify and evaluate the efficacy of wireless sensors in restorative dentistry.

## 2. Materials and Methods

### 2.1. Protocol and Registration

The search approach utilized in this systematic review adhered to the PRISMA (Preferred Reporting Items for Systematic Reviews and Meta-Analyses) guidelines [15]. The protocol was formally recorded on the Open Science Forum Database and can be accessed using the following link: https://doi.org/10.17605/OSF.IO/8M5F6 (accessed on 29 February 2024).

### 2.2. Eligibility Criteria

The systematic review was based on a question formulated using the Population, Intervention, Comparison, and Outcomes (PICO) framework:

P: Studies involving the application of wireless sensors in restorative dentistry.
I: Utilization of wireless sensors in restorative dentistry procedures.
C: Comparative control experiments.
O: Efficacy of wireless sensors.

Conferences, editorials, abstracts, systematic and narrative reviews, meta-analyses, and investigations that lacked critical details about fabrication and methods were excluded.

### 2.3. Information Sources

The search strategy utilized prominent scientific databases, including PubMed/MEDLINE, SCOPUS, and SCIELO, supplemented by exploration of grey literature sources via Google Scholar. An extensive electronic database search was conducted from the inception of these databases until February 2024, without language limitations. Additional records were identified through scrutiny of the reference lists and citations within all selected full-text records eligible for inclusion in the systematic review.

### 2.4. Search Strategy

The search approach included the use of the following search terms: “wireless sensor”, OR “wireless communication system”, OR “sensors”, OR “biosensor”, “3D sensors”, OR “lab on a chip”, OR “micro physiological systems”, OR “bioassays”, AND “restorative dentistry”, AND “dentistry”. This method was initially devised for PubMed and later modified for use with other databases.

Two scholars individually evaluated titles and abstracts for eligibility, followed by a thorough examination of full-text articles. Suitability founded on full-text valuation was independently determined by each author, with any discrepancies decided through consensus. Interobserver agreement was assessed using the Kappa statistical test with a threshold of >90 to establish statistical significance.

### 2.5. Data Collection

Two researchers individually collected records utilizing adaptable information extraction techniques. The collected data underwent a comparative analysis to ensure consistency. The data included information on the application of wireless systems, as well as key elements, such as the materials used in their construction and important research outcomes. Additionally, the authors’ names and publication years were documented.

### 2.6. Evaluation of Bias Risk and Research Quality in Specific Studies

To evaluate the methodological rigor of the included research, the 16-item quality assessment tool (QATSDD) was employed [16]. This instrument consists of 16 criteria, covering an “explicit theoretical framework; a statement of objectives, a precise depiction of the research environment, substantiation of sample size adequacy, inclusion of a representative sample, a thorough account of the data collection procedure, a rationale for the chosen data collection tool, comprehensive recruitment data, a statistical evaluation of the reliability and validity of the measurement tool, alignment between the stated research question and the method of data collection, coherence between the research question and the format and content of data collection, harmony between the research question and the method of analysis, a well-founded justification for the selected analytical method, an evaluation of the reliability of the analytical process, demonstration of user involvement in the design, and a critical discussion of strengths and limitations”. Each aspect reviewed carries equal weight. Each criterion is assigned a value ranging from 0 to 3 (0 = insufficient detail to inform a decision; 1 = inadequately provided; 2 = moderately provided; 3 = comprehensively provided). The cumulative score of these criteria yields a total result for the body of evidence, expressed as a proportion of the maximum attainable score.

### 2.7. Summary Measurements

Descriptive measures, such as mean differences and standard deviations, were utilized to obtain data from the included studies for continuous outcomes. In instances where the encompassed papers revealed a relative amount of homogeneity, the possibility of conducting a meta-analysis was evaluated as a probable next phase.

This study did not necessitate ethical authorization.

## 3. Results

### 3.1. Study Selection

**Figure 1:**
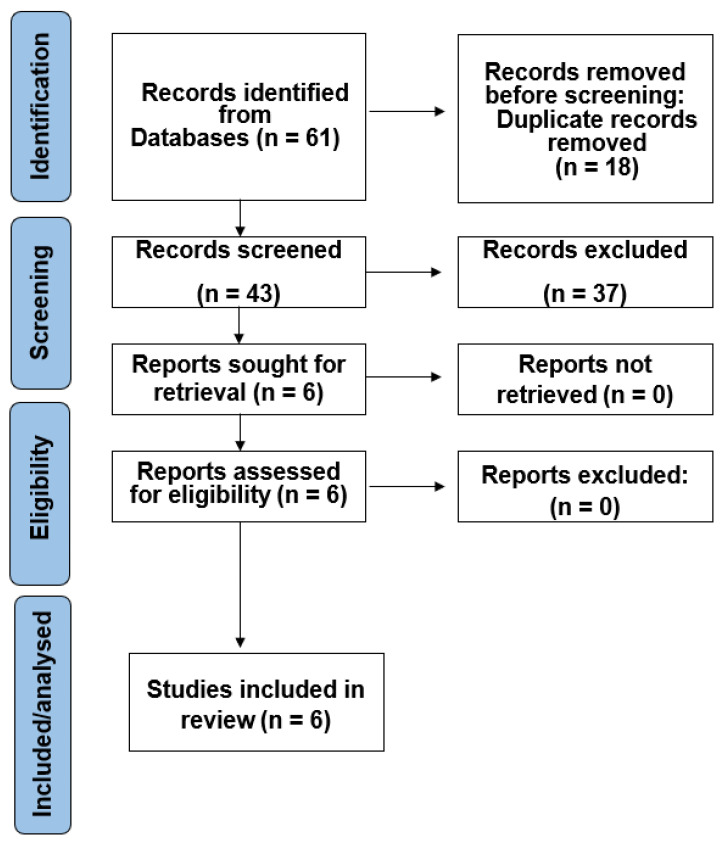
Selection method outline.

After conducting the search method, 61 studies were identified in electronic databases. Following the removal of duplicate entries and application of eligibility criteria, 43 articles underwent a comprehensive full-text appraisal. The exclusion of reports through the full-text revision was primarily due to a lack of focus on wireless sensors. Following the final phase of evaluating eligibility, the systematic review included six papers. Figure 1 illustrates a detailed search flowchart.

### 3.2. Features of the Studies

Table 1 summarizes the descriptive features of the six reports included in this study. The publications covered in this review span from 2007 to 2022 [7,12]. Five experiments were conducted in vitro [7,8,9,10,12], while one was a randomized clinical trial [11]. Most of the studies were carried out in Asia [7,8,10], with two in Brazil [9,12] and another in the United States [11]. The investigations focused on wireless sensors for cavity diagnosis [8,9,12], toothbrush forces [11], facial mask applications [7], and physiological parameter detection from dental implants [10].

### 3.3. Main Outcomes

Table 2 displays the features of the wireless sensor used in each investigation. All wireless sensors demonstrated efficacy in achieving the objectives established by each study and demonstrated the validity, accuracy, and reproducibility of the wireless sensor.

The three studies that evaluated the diagnosis of caries demonstrated effective results in its detection [8,9,12]. Tabata et al. [8] found a strong correlation between caries’ pH readings and Raman capacities. “The wireless module with Ir/IrOx pH electrode showed a sensitivity of −54.9 mV/pH and a potential drift of 0.18 ± 0.1 mV/min, which are suitable for chairside pH measurement equipment”. Additionally, “the average pH values of non-caries and caries areas assessed with the manufactured wireless pH sensor were 6.9 and 5.8, respectively, consistent with the wired pH sensor results”. Importantly, by designing the wireless pH sensor to resemble a “dental explorer”, it may notice cavities in the mouth without injuring the tooth surface. Melo et al. [9] reported that for the “wireless digital system, an exposure duration of 0.25 s yielded the highest accuracy result, with mean ROC curve areas ranging from 0.53 to 0.62. The time range of 0.06 s to 0.20 s was deemed an appropriate interval of exposure time for caries diagnosis utilizing the wireless system, with no statistical difference compared to the 0.25 s exposure time. According to the authors, reducing the exposure duration reduces the patient exposure dose, and this decrease cannot be achieved at the expense of image quality. In an in vitro setting with standardized conditions, Haiter-Neto et al. [12] obtained “radiographs of 160 non-cavitated proximal surfaces using the Digora FMX, Digora Optime, Schick CDR, and Schick CDR wireless systems”. Eight scholars assessed “proximal carious lesions on a five-point confidence scale”. The presence of caries was confirmed histologically. The authors demonstrated that the Digora Optime and CDR wireless systems had the highest sensitivity values. The difference in sensitivity between the two Digora systems was statistically significant, with the Digora Optime system showing higher sensitivity than the Digora FMX system (*p* = 0.008). The sensitivity of the CDR Wireless system was also significantly higher than that of the Digora FMX system, but it was not significantly higher than that of the Schick CDR system (*p* = 0.13). The overall accuracy of the digital radiography systems varied, ranging from 0.61 for the Digora Optime system to 0.65 for the wireless system.

Li and Lu proposed a miniature intra-oral dental implant system with an integrated biosensor device [10]. The dental implant system, or platform, is positioned over the maxilla, enabling minimally invasive treatments and a unique biosensing method for human blood analysis. The dental implant system consists of an implant fixture, a prosthetic abutment, a biosensor module, a Bluetooth 4.0 wireless module, and a direct current button cell battery. The electrochemical biosensor comprises three electrodes: working, reference, and counter electrodes, which are designed to pass through the titanium implant fixture beneath the biosensor module. These electrodes are exposed to the blood pool within the maxillary bone marrow, where they undergo oxidation/reduction reactions with a biosensing enzyme coating. To validate the planned proposal, “the glucose oxidase (GOD) enzyme” immobilization method and “in vitro” glucose level findings were successfully conducted, with the glucose biosensor system demonstrating confirmed sensitivity, linearity, and repeatability. Furthermore, a pilot canine animal exemplar using the novel channel demonstrated remarkable concordance with the classic method of blood sugar monitoring via dermal pricks.

Lin et al. [7] developed a “wireless sensor-integrated face mask” utilizing augmented reality technology. Augmented reality technology enables the overlaying of virtual 3D objects onto real-world environments and connections with tangible pieces to generate required meanings. The augmented reality method facilitated precise estimation of the mask’s size, which was then created via 3D printing. Body temperature and breathing sensors were integrated within the mask, enabling continuous monitoring of important human body parameters without the requisite to subtract individual protective apparatus. Additionally, the mask’s exterior layer is composed of modified conductive fabric, which enhances airborne aerosol filtration. Upon operating an external voltage to conductive fabrics, the filtering competence of particulate material 2.5 increased significantly. A smartwatch with a heart rate sensor was paired with the mask, allowing sensor data to be wirelessly displayed.

Janusz et al. [11] investigated the capability of a power brush with a wireless display to increase brushing strength and care. At baseline, 61 pre-screened respondents were videotaped cleaning their teeth. The wireless display was also videoed. The participants were then randomly assigned to either the power brush alone or the power brush with a wireless display. After 30 days of home use, respondents returned to brush for two minutes in front of a two-way mirror. The brushing behavior and wireless display were videotaped. The pressure sensor analysis involved fifty-eight participants. The reduction in pressure sensor activation time at day 30 compared to baseline was 88.5% for the power brush with wireless display and 53.4% for the power brush alone (*p* < 0.05). Thus, participants utilizing the power brush with the wireless display applied less force during brushing compared to those using only the power brush.

The investigations also included rigorous validation processes for the wireless sensor to ensure accuracy, precision, and reliability in the tested trials. These approaches included repeated tests, mathematical computations, and previous experiments.

### 3.4. Results’ Synthesis

The systematic review did not justify a meta-analysis due to significant variations in the methodological approaches and research designs among the included studies. Several foundational references for outcome evaluation, as well as the consideration of different mechanisms, were evident across wireless sensors of varying designs. Therefore, the analysis was limited to a qualitative assessment.

### 3.5. Bias Risk and Study Quality in Individual Studies

All studies included in this assessment met at least 75% of the established quality criteria [16], classifying them as of good quality (Table 3). Most of these studies lacked sample size computation (criterion 4) and the inclusion of a representative sample (criterion 5). However, it is relevant to comment that the remaining criteria reviewed by the evaluation method were met.

## 4. Discussion

This study was the first to assess the utilization and efficacy of wireless sensors in restorative dentistry practice. Despite the differences in objectives and methodological approaches among the studies examined, all of them demonstrated the efficacy of the analyzed mechanisms, which were supported by appropriate validation processes.

The validation processes in Lin et al.’s study [7] included verifying the precision of size estimation and alignment by comparing augmented reality-generated measurements with physical measurements of the mask, ensuring accurate size estimation and alignment of virtual objects with real-world objects, validating sensor accuracy by comparing measurements obtained from the integrated sensors with measurements from established reference devices under various conditions, calibrating sensors to ensure accurate measurement of vital parameters (temperature and respiratory rate), applying external voltage to the conductive textiles and measuring changes in filtration efficiency, comparing results with baseline measurements without voltage application, and testing data transmission under different scenarios (e.g., varying distances between the mask and smartwatch, and the presence of interference) to assess performance. In the study by Tabata et al. [8], these processes included comparing Raman reading and pH reading methods for evaluating dental caries, assessing the consistency and reliability of results obtained from both methods to ensure accurate detection of dental caries, developing and calibrating an Ir/IrOx pH sensing probe for quantitative evaluation of dental caries, developing a LabVIEW-based real-time data monitoring program for the wireless pH sensor, determining the accuracy and stability of the wireless pH sensor, and measuring pH on the dental caries’ surfaces using the wireless pH sensor. Melo et al. [9], on their part, compared radiographic scores assigned by four observers for the presence of carious lesions on proximal tooth surfaces against histological sections of the teeth to assess the system’s ability to accurately detect caries. Additionally, they evaluated the accuracy of caries’ detection using the wireless system. Furthermore, they determined the area under the ROC curve (Az) for each exposure time to evaluate the system’s diagnostic accuracy in detecting carious lesions and analyzed pixel intensity values to evaluate image quality. Li et al. [10] validated the immobilization process of the glucose oxidase (GOD) enzyme by assessing the success of the GOD enzyme immobilization onto the biosensor module to ensure the proper functioning of the biosensor system. Additionally, they tested the biosensor system’s ability to accurately detect glucose levels in vitro, ensuring sensitivity, linearity, and repeatability of measurements, and validated the biosensing pathway using a preliminary canine animal model. Januz et al. [11] compared the thoroughness of brushing across different tooth surfaces (dentition and lingual/buccal) between baseline and day 30 for subjects using the power brush alone and those using the power brush with the wireless display. They also assessed the ability of the power brush with a wireless remote display to improve brushing thoroughness. Finally, Haiter-Neto et al. [12] validated the accuracy of radiographic imaging for detecting proximal carious lesions. They obtained radiographs of non-cavitated proximal surfaces using different intra-oral digital systems under standardized conditions. Additionally, they validated observer agreement in recording proximal carious lesions and confirmed the presence of caries using histological examination as the gold standard to ensure the accuracy of caries’ detection by the intra-oral digital systems.

Wireless communication, when paired with sensors, is a promising technique for creating small, highly functional devices for various applications. As observed in this systematic review, one study integrated a miniature “pH electrode with Ir/IrOx as a sensing material with a wireless module” [8]. “The pH electrode’s shape and size were designed to resemble that of a dental explorer. The Ir/IrOx pH electrode, coupled to a gold wire, is approximately 2 cm long. The sensor measures 135 mm in length, 25 mm in width, and 25 mm in height. It weighs approximately 30 g”. “The pH sensor is easily held in one hand, and the entire system is suitable for use in a dental clinic. The mechanism includes a low-cost wireless communication module that may be compatible with the Internet of Things. The pH sensitivity of the wireless pH sensor was tested in seven pH calibration solutions ranging from 3 to 9”. After adjusting the pH, the potential response stabilized within a few seconds, demonstrating the system’s robustness [8]. The pH profiles of the “non-caries and caries sections” followed similar trends as in a prior study [17]. However, the surface of a cavity has a substantially higher proton concentration, resulting in a lower pH than the surrounding cavity area [8]. In clinical practice, dentists diagnose each carious lesion based on subjective visual examination, intra-oral radiographs, and patient feedback [18]. Nevertheless, conventional techniques do not offer quantifiable, immediate findings. “Intra-oral pH measurement of the dental caries surface” can be used to diagnose caries activity in real-time, as well as to follow up on non-surgical management and monitor arrested injuries [8].

Solid sensor characteristics require more radiographic exposures to capture the same amount of data as traditional film and phosphor-stimulable plates [19]. When examining the overall image quality of two wired system versions subjectively, Kitagawa et al. [20] “varied exposure lengths from 0.05 to 0.40 s, with the best results obtained with exposure times of 0.09 s for one version and 0.12 s for the other”. These outcomes indicate that comparable procedures may have varying optimal exposure times. In the study reviewed in this systematic review [9], the wireless version was assessed utilizing histological corroboration, and the exposure time that produced the best outcomes was 0.25 s (15 i), with an adequate time exposure variety of 0.06–0.20 s (4–12 i), which was similar to the results found for one wired version.

When comparing several digital techniques in the recognition of proximal caries, another study reviewed here [12] used a wireless system and selected an exposure period of “0.22 s for the premolar region and 0.26 s for the molar region, achieving an accuracy value of 64%”. The selected exposure intervals, as well as the accuracy value, are similar to those that produced the greatest results in Melo et al.’s study [9]. This digital system’s wireless connection, which transmits gathered data via radiofrequency waves, is a key feature. When the wireless digital receptor is exposed to radiation, the electrical charge generated by the silicon crystals transforms into radiofrequency waves. These waves are intercepted by the system’s base station antenna and transformed into binary units, which are then transmitted to the computer via a fiber optic cable. This system incorporates a non-rechargeable battery for data transfer to the base station antenna [9,12]. It is important to note that these in vitro experiments enabled histologic analysis of the approximal surfaces as the final validation criterion.

The redesigned micro-intra-oral dental implant system with an integrated biosensor device includes a prototype that is roughly twice the size of a comparable unit already in use for clinical purposes [10]. This increase in size is aimed at addressing existing challenges, such as designing and manufacturing compact components, immobilizing the “GOD enzyme on fine electrodes”, and assembling the method. Cyclic voltammetry and the resulting Bluetooth signals demonstrated reasonable linearity with glucose levels. For every 2 mg/dL increase in glucose level, the current increased by about 1 μA, equivalent to 0.8 reading units of the Bluetooth transmission. Moreover, the GOD enzyme performed well in the laboratory with pure glucose solutions. However, it has been reported that the GOD enzyme degrades after 48 h, resulting in a decreased signal responsiveness over time [21]. This challenge becomes more complex when the biosensing platform is intended for extended use, such as 30 days. It is important to note that the projected proposal is most appropriate for individuals who require regular invasive procedures. For example, people with type I diabetes mellitus need to observe their blood sugar levels four times a day, and subjects undergoing hemodialysis can have a comparable challenge [22]. When patients undergo frequent invasive procedures, the proposed device may help alleviate their discomfort [10].

Regarding the revised “wireless sensor-integrated face mask” using increased reality machinery [7], certain aspects must be emphasized. To exhibit physical information and incorporate real-time sensors, the smartwatch was intended to work with “the sensor-integrated mask”. The wristwatch includes “a microcontroller, a wireless transmission module, a photoplethysmography heart rate sensor, and an organic light-emitting diode display”. “The smartwatch could display body temperature and respiration rate data from the temperature and respiratory sensors implanted in the face mask via the wireless transmission module”. The heart rate sensor was developed using the Max30102 module to measure photoplethysmography on the skin surface, which offers the advantages of portability, compactness, and durability. Photoplethysmography provides valuable information about the human cardiovascular system [23]. Photoplethysmography employs low-intensity infrared light from light-emitting diodes, which is absorbed by bones, skin pigments, venous blood, and arterial blood [24]. The photoreceptor then converts the photonic signal into a voltage. The sensor-integrated mask system serves as the first line of defense against global threats posed by pathogens and air pollutants [7]. The outcomes of this investigation hold significant implications, particularly considering recent discoveries. These findings revealed that the internal and external strata of the masks were found to contain 3.3 × 10^8^ and 8.5 × 10^8^ colony-forming units, correspondingly. Moreover, the infectivity proportions of the masks demonstrated an upward trend in tandem with the duration of mask-wearing [25].

A study reviewed in this systematic review examined the capability of a power brush with a wireless system to enhance the brushing force and thoroughness [11]. This study evaluated the effects of the most recent innovation in oscillating–rotating power toothbrushes, a power brush with a wireless remote display, versus the power brush without a wireless display. The brushing period, stress, procedure, and bristle arrangement are all factors that influence the amount of plaque removed by a toothbrush. Another important factor is the brush’s motion, or lack thereof. Power toothbrushes with an oscillating–rotating action were found to reduce significantly more plaque and gingivitis than manual toothbrushes [26]. In contrast to the power brush alone, the power brush equipped with a wireless display improved brushing thoroughness and uniformity. Additionally, it significantly minimized the variation in brushing duration among the four mouth quadrants [11].

Wireless sensors in restorative dentistry offer several advantages, including mobility, convenience, real-time monitoring, integration, and patient comfort [27]. However, they also have limitations, such as power consumption, interference, range, data security, and cost. Therefore, it is essential to consider these factors when evaluating the use of wireless sensors in dental practice. A further limitation encountered in this systematic review arose from the inability to perform a quantitative analysis. This restriction largely resulted from the methodological and design differences noted in the studies reviewed. These studies encompassed a range of research designs, employed numerous important references for outcome evaluation, and investigated various wireless sensor-utilizing devices with differing designs.

In addition to the current advantages and limitations of wireless sensors in restorative dentistry, it is also pertinent to consider the future trends in this field, particularly regarding the integration of artificial intelligence. Artificial-intelligence-based wireless sensors hold promising potential to revolutionize dental practice by offering advanced capabilities, such as predictive analytics, personalized treatment planning, and automated monitoring systems. By leveraging machine learning algorithms, these sensors can analyze vast amounts of patient data in real time, allowing for more accurate diagnoses, treatment recommendations, and outcome predictions. Furthermore, artificial-intelligence-driven wireless sensors have the potential to enhance the efficiency and precision of restorative procedures, leading to improved clinical outcomes and patient satisfaction.

## 5. Conclusions

The reports revised in this systematic review illustrate the potential of wireless sensors in restorative dentistry, especially in the areas of caries’ detection, dental implant systems, face masks, and power brushes. These technologies hold promise for enhancing patient outcomes and alleviating the workload of dental practitioners.

## Figures and Tables

**Table 1:** Descriptive characteristics of the studies included.

Authors and Publication Year	Country	Study Design	Main Aim
Lin et al., 2022 [7]	Taiwan	In vitro	A wireless sensor-integrated face mask was created with Au@SnO_2_ nanoparticle-modified conductive structures, utilizing amplified reality machinery.
Tabata et al., 2021 [8]	Japan	In vitro	A wireless caries sensing tool, comparable in size to a dental explorer, was designed to compare two sensing modalities: Raman readings and pH readings, for the evaluation of tooth decay.
Melo et al., 2019 [9]	Brazil	In vitro	A wireless system was employed to assess the impact of dissimilar exposure durations on caries’ detection and pixel intensity values.
Li et al., 2015 [10]	Taiwan	In vitro	A novel “miniature intra-oral dental implant system”, which incorporates a “built-in biosensor device”, is being suggested. The implant fixture’s placement enables “continuous blood analysis and management via the maxillary bone marrow, facilitated by the dental implant platform”.
Janusz et al., 2008 [11]	USA	Randomized clinical trial	This report assessed the efficacy of a “power brush” equipped with a “wireless remote display” in enhancing brushing force and thoroughness.
Haiter-Neto et al., 2007 [12]	Brazil	In vitro	In this study, the accuracy of two intra-oral digital systems for the radiographic identification of proximal carious lesions was evaluated.

**Table 2:** Main findings.

Authors	Sensor Characteristics and Operation	Main Results
Lin et al. [7]	The mask’s interior contained an electronic microprocessor with two sensors: a temperature sensor and a respiration sensor. These sensors monitored “body temperature and respiratory rate”, respectively. The microcontroller then combined the data and wirelessly communicated it to the smartwatch.	The “intelligent mask” not only enhances security by incorporating “conductive textiles and nanoparticles”, but it also integrates a variety of sensor systems for real-time monitoring without requiring the removal of the mask.
Tabata et al. [8]	The measurement system circuit included an analog–digital converter (ADC), a “wireless module, and a 3.3 V lithium-ion battery”. The pH conductor and reference electrode were directly connected to the ADC component. All components were housed “in a 3D-printed PLA polymer” casing. “The wireless sensor’s pH” reaction was evaluated with seven pH solutions (pH 3 to pH 9). “The wireless module’s software was written in Arduino, and real-time data collection was done using a LabVIEW-based application”.	“The pH of the dental caries surface was gauged with a wireless pH sensor”, revealing pH values of 6.9 and 5.7 in non-caries and caries areas, respectively. This wireless pH sensor could prove beneficial in comprehending dental caries conditions and aiding dentists in selectively removing caries while maintaining the non-caries structure.
Melo et al. [9]	“The data collected are transmitted through radiofrequency waves. When the wireless sensor’s digital receptor is exposed to radiation, the electric charge generated by the exposure of the silicon crystals is converted into radiofrequency waves. These waves are captured by the system’s base station antenna and converted into binary units, which are then transmitted to the computer via a fiber optic cable”.	“The wireless sensor was evaluated with histological confirmation, and it was determined that the optimal exposure time for caries diagnosis was 0.25 s (15 i), with an acceptable range of 0.06–0.20 s (4–12 i)”.
Li et al. [10]	“The dental implant system comprises an implant fixture, a prosthetic abutment, a biosensor module, a Bluetooth 4.0 wireless module, and a DC button cell battery. The electrochemical biosensor features three electrodes: working, reference, and counter, which are positioned to penetrate the titanium implant fixture beneath the biosensor module. These electrodes are exposed to the blood pool within the maxillary bone marrow and engage in oxidation/reduction reactions with the biosensing enzyme coating”.	To validate the planned proposal, the restriction course of glucose oxidase (GOD) enzyme is successfully executed, and in vitro glucose level detections are performed, demonstrating “the sensitivity, linearity, and repeatability of the glucose biosensor system. Additionally, a preliminary canine animal model utilizing the new pathway” exhibits substantial agreement with the conventional technique of blood sugar detection via dermal pricks.
Janusz et al. [11]	“The wireless display and toothbrush” interconnect through an automatic chip embedded in the toothbrush handle. The display can be situated up to 10–15 feet from the patient, enabling them to conveniently “view the two-minute timer, brushing mode, quadrant timer, and pressure signal, which illuminates when a force of over 3 N is exerted”.	Following 30 days of at-home use, participants utilizing the power brush equipped with a wireless display experienced a reduction in pressure sensor activation time by 88.5% compared to the baseline, while those using the power brush alone saw a reduction of 53.4% (*p* = 0.034). Individuals using the power brush with the wireless display applied less force than those using the power brush alone.
Haiter-Neto et al. [12]	The data collected “are transmitted via radiofrequency waves”. When the digital receptor of the wireless sensor is “exposed to radiation, the electric charge generated by the silicon” crystals’ exposure is converted into radiofrequency waves. These waves are captured by the “system’s base station antenna and converted into binary units, which are then transmitted to the computer via a fiber optic cable”.	The wireless sensor digital systems exhibited significantly higher sensitivities compared to their precursors. The investigators noted no substantial differences in “specificity among the Digora FMX, Schick CDR, and CDR wireless systems, all of which had significantly higher specificity than the Digora Optime system (*p* < 0.02). The positive predictive value for the Digora Optime system was influenced by its high sensitivity and low specificity, resulting in a lower value compared to the two CDR systems (*p* < 0.02)”.

**Table 3:** Bias risk and study quality in individual studies [15] *.

Study	Criteria Completely Satisfied	Percentage Score of Compliance
Lin et al., 2022 [7]	15	94%
Tabata et al., 2021 [8]	14	88%
Melo et al., 2019 [9]	14	88%
Li et al., 2015 [10]	14	88%
Janusz et al., 2008 [11]	14	88%
Haiter-Neto et al. [12]	15	94%

* The instrument comprises 16 criteria: (1) explicit theoretical framework, (2) a statement of objectives, (3) a precise depiction of the research environment, (4) substantiation of sample size adequacy, (5) inclusion of a representative sample, (6) a thorough account of the data collection procedure, (7) a rationale for the chosen data collection tool, (8) comprehensive recruitment data, (9) a statistical evaluation of the reliability and validity of the measurement tool, (10) alignment between the stated research question and the method of data collection, (11) coherence between the research question and the format and content of data collection, (12) harmony between the research question and the method of analysis, (13) a well-founded justification for the selected analytical method, (14) an evaluation of the reliability of the analytical process, (15) demonstration of user involvement in the design, and (16) a critical discussion of strengths and limitations.

## Data Availability

The data obtained in this review were pooled from the included investigations.
